# Enhancing the Hydrophobicity and Antibacterial Properties of SiCN-Coated Surfaces with Quaternization to Address Peri-Implantitis

**DOI:** 10.3390/ma16175751

**Published:** 2023-08-22

**Authors:** Chao-Ching Chiang, Xinyi Xia, Valentin Craciun, Mateus Garcia Rocha, Samira Esteves Afonso Camargo, Fernanda Regina Godoy Rocha, Sarathy K. Gopalakrishnan, Kirk J. Ziegler, Fan Ren, Josephine F. Esquivel-Upshaw

**Affiliations:** 1 Department of Chemical Engineering, College of Engineering, University of Florida, Gainesville, FL 32611, USA; cchiang@ufl.edu (C.-C.C.);; 2 National Institute for Lasers, Plasma and Radiation Physics, RO-077125 Magurele, Ilfov, Romania; 3 Department of Restorative Dental Sciences, Division of Operative Dentistry, College of Dentistry, University of Florida, Gainesville, FL 32610, USA; 4 Department of Comprehensive Oral Healthy, Adams Dental School, University of North Carolina, Chapel Hill, NC 27599, USA; 5 Department of Periodontology, College of Dentistry, University of Florida, Gainesville, FL 32610, USA; 6 Department of Restorative Dental Sciences, Division of Prosthodontics, College of Dentistry, University of Florida, Gainesville, FL 32610, USA

**Keywords:** peri-implantitis, silicon carbonitride (SiCN), quaternization

## Abstract

Peri-implantitis is a major cause of dental implant failure. This disease is an inflammation of the tissues surrounding the implant, and, while the cause is multi-factorial, bacteria is the main culprit in initiating an inflammatory reaction. Dental implants with silicon carbonitride (SiCN) coatings have several potential advantages over traditional titanium implants, but their antibacterial efficiency has not yet been evaluated. The purpose of this study was to determine the anti-bacterial potential of SiCN by modifying the surface of SiCN-coated implants to have a positive charge on the nitrogen atoms through the quaternization of the surface atoms. The changes in surface chemistry were confirmed using contact angle measurement and XPS analysis. The modified SiCN surfaces were inoculated with *Streptococcus mutans* (*S. mutans*) and compared with a silicon control. The cultured bacterial colonies for the experimental group were 80% less than the control silicon surface. Fluorescent microscopy with live bacteria staining demonstrated significantly reduced bacterial coverage after 3 and 7 days of incubation. Scanning electron microscopy (SEM) was used to visualize the coated surfaces after bacterial inoculation, and the mechanism for the antibacterial properties of the quaternized SiCN was confirmed by observing ruptured bacteria membrane along the surface.

## 1. Introduction

Dental implants have revolutionized modern dentistry by providing an effective alternative for tooth replacement. According to a recent comprehensive analysis and meta-analysis, dental implants have a potential 10-year survival rate of up to 96.4% [1]. However, peri-implantitis has emerged as a significant concern in the practice of implant dentistry. Peri-implantitis is usually characterized by inflammation and bone loss, which ultimately lead to implant failure [2,3]. Increased implant failure rates result in significant morbidity and increased healthcare costs [4,5,6,7]. Conventional treatment approaches, including mechanical debridement and antimicrobial therapy, have limitations in effectively managing peri-implantitis [8,9,10,11]. Prevention strategies that focus on reducing bacterial colonization and biofilm formation on the implant surface are considered critical factors in the development and progression of peri-implantitis [12,13]. 

In recent years, the surface modification of implant materials has emerged as a promising strategy to enhance the antibacterial ability of implants and mitigate the risk of peri-implantitis. The surface properties of implants, such as roughness, hydrophobicity/hydrophilicity, and charge, can be altered to influence bacterial adhesion, colonization, and biofilm formation [14,15,16]. Various surface-modification techniques, including physical, chemical, and biological approaches, have been investigated to improve the antibacterial ability of implants. For example, Harris et al. utilized poly(L-lysine)-grafted poly(ethylene glycol) (PLL-g-PEG) to create an antiadhesive surface on titanium which reduced 90% of Staphylococcus aureus on the modified surface [17]. Nishida et al. developed a surface modification using carboxymethyl betaine (CMB) to create an antifouling surface, highlighting the potential of zwitterionic polymers as a viable option for the surface modification of dental implants [18]. In our prior research, we conducted numerous investigations to confirm the viability of utilizing silicon carbide (SiC) coating on dental implants. This involved studying the cytotoxicity and biocompatibility of this coating using human osteoblasts [19], as well as demonstrating SiC’s corrosion protection capabilities [20]. Additionally, the simulation of installing coated implants into human bone [21], along with the potential of SiC coating for cell proliferation and mineralization, have also been demonstrated [19].

Aside from the various methods mentioned earlier for modifying the surface of implants, research has shown promising bactericidal effects from surfaces containing quaternized nitrogen, where positively charged nitrogen atoms take the leading role [22,23,24,25]. It has also been demonstrated that the quaternization of a surface coating can be a successful approach in enhancing the antibacterial characteristics of the implant surface [26,27,28,29]. The majority of bacterial cellular membranes possess a negative charge, making them susceptible to being targeted by cationic biocides [30,31,32]. As a result, molecules containing nitrogen atoms in a quaternary state have been observed to disrupt the cell wall of bacteria, causing the leakage of cell contents and eventual cell death through apoptosis [33,34].

On the other hand, decreasing the chance for bacteria biofilms to form on dental implants is also the main means of preventing the onset of peri-implantitis, since certain bacteria must inhabit the dental implant first [35,36,37]. Before the microbiota undergoes a transition to the late stage, *S. mutans* play a significant role in the formation of oral bacterial biofilms during their early stages [38,39,40]. DNA Pyrosequencing of plaque samples from peri-implantitis patients revealed *S. mutans* as one of the predominant species in the associated biofilms [41]. Various studies have also been conducted to evaluate the connections between *S. mutans* and peri-implantitis, from its adherence, viability, and migration on implant surfaces to biofilm analysis after different treatments [37,42,43]. In addition, due to its characteristic of being a facultative anaerobe organism, *S. mutans* is also a perfect candidate to mimic the environment of both the related aerobic and anaerobic bacteria causing peri-implantitis [44].

The aim of this study was to quaternize SiCN coating and determine the potential of this modified quaternized SiCN (QSiCN) coating for increased hydrophobicity and antibacterial properties against *S. mutans*. Contact angle measurements were employed to assess the enhanced water repellency resulting from the quaternization process, while X-ray photoelectron spectroscopy (XPS) analysis was performed to characterize the modified coating surfaces. Bacterial culture experiments were conducted to confirm the reduction in bacterial activity, as indicated by colony counts. Fluorescent and scanning electron microscopy images were utilized to investigate the extent of bacterial coverage on the surfaces and to explore evidence of membrane damage to bacterial cells. The ultimate goal is to clarify the underlying mechanism that makes the antibacterial coating effective and to establish a foundation for its potential utilization in clinically preventing peri-implantitis. 

## 2. Materials and Methods

The sample preparation begins by depositing a layer of SiCN, 100 nm in thickness, on a silicon wafer through the use of Plasma Enhanced Chemical Vapor Deposition (PECVD) at a pressure of 900 mTorr, utilizing a processing gas mixture of silane, methane, helium, and ammonia. The concentration of nitrogen in the SiCN layer was varied through adjustments in the flow rate of ammonia and had previously been analyzed using XPS analysis [45]. After the SiCN layer was applied, the samples were cut into 1 cm squares and rinsed with acetone and IPA. The nitrogen atoms on the SiCN surface were then converted to quaternary nitrogen by immersing the samples in a solution of acetonitrile and allyl bromide for one hour, through a process known as the Menschutkin reaction. Following quaternization, the samples were rinsed with isopropanol and deionized water to remove any excess solvent and reagent. 

A sessile contact angle measurement was used to reveal the surface wettability of the samples. A 3 μL droplet of DI water was dropped on the surface by a syringe, and an image was captured by a microscopic camera with a cold backlit light source to prevent heating up the sample and the water. Analysis using the Young–Laplace equation was applied to the images to calculate the fitted contact angle. The contact angle data for each individual sample were determined as the average of five measurements. The chemical composition of the deposited films was studied using X-ray Photoelectron Spectroscopy (XPS) with an ESCALAB 250Xi instrument (Thermo Fisher Scientific, Pittsburgh, PA, USA) equipped with a monochromatic aluminum anode as the X-ray source. High-resolution scans for detailed peak analysis were performed at an electron pass energy of 20 eV and an energy step size of 0.1 eV, with a scanning range focusing on the nitrogen 1 s region. To better target the quaternized nitrogen atoms on the surface itself, scans were acquired at 0° and 45° tilting with respect to the normal. New spectra were also acquired after a gentle 300 s sputtering with an Ar cluster and then with 500 eV Ar ions to remove a layer of atoms on the surface and validate the location of the quaternized nitrogen atoms.

**Figure 1:**
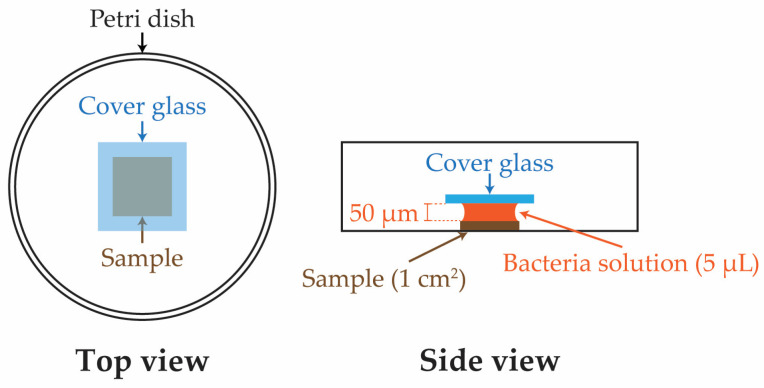
Schematic drawings of sample testing configuration for bacterial culture.

The bacterial culture was prepared by thawing and centrifuging a frozen stock of *S. mutans* to obtain pellets. These pellets were resuspended in a liquid growth media of Brain Heart Infusion broth (Himedia, Mumbai, India). The bacterial solution was allowed to incubate for 24 h at 37 °C in an incubator before being diluted to an optical density of 650 under a 650 nm wavelength using a spectrophotometer. The resulting solution contained approximately 10^7^ colony-forming units per milliliter (CFU/mL) of bacteria. Before growing live bacteria, 6 (six) replicates of each condition—Si, SiCN, and quaternized SiCN samples with 5, 10, and 15% nitrogen content—were decontaminated by rinsing and soaking in an ethanol bath for 20 min. A set of SiC samples was also added to the experiment as a comparison. Each sample was then placed in a separate sterile petri dish. Subsequently, the bacteria solution with 10^7^ CFU/mL of *S. mutans* was applied onto the surface of the Si, SiC, SiCN, or QSiCN substrates using a micro-pipette, and this was followed by covering the sample surface from the top with another sterile cover glass slide. The gravitational force created by the glass slide allowed the bacteria solution to spread evenly up to the edge of the sample. In this case, the solution would not extend beyond the edge of the sample due to its surface tension (Figure 1). Thus, the thickness of the biofilm was controlled by the volume of the bacteria solution (5 μL), since the area of the sample was the same (1 cm^2^). The whole container was then incubated at 37 °C for 24 and 48 h. Subsequently, the content of the container was transferred to a 50 mL falcon tube that contained 5 mL of BHI solution. The falcon tube was vortexed to peel off the biofilm and disperse the bacteria evenly in the solution. The resulting solution was then serially diluted with 10 and 100 fold dilution by BHI medium on a 24-well sterile plate. Next, 100 mL of the solution was dropped on a BHI agar plate and spread with an L-shaped plastic cell spreader, followed by incubation at 37 °C for 48 h. The colonies on the plates were then counted, multiplied back to the unit of CFU/mL and recorded for each sample accordingly.

For the antibacterial activity analysis, three samples of each of the SiCN samples with 5%, 10%, and 15% nitrogen content and a Si control group were placed in a sterilized 24-well plate and sterilized using ethanol. The samples were then rinsed three times with a solution of 1X phosphate-buffered saline (PBS). A total of 50 µL of 10^7^ CFU/mL standardized bacterial inoculum and 1 mL of BHI broth culture media were added to the plate, covering up the samples. The plate was placed in a shaking incubator set at 37 °C and 75 rpm for 90 min to allow the bacteria to initially adhere to the samples in the form of biofilms. The supernatant was discarded, and the fresh broth was added to the plate, which was incubated for an additional 3 and 7 days to allow the biofilms to develop. After the designated time period, the samples were carefully rinsed with PBS and treated with formaldehyde for 15 min. The effectiveness of the Si and QSiCN samples in inhibiting bacterial growth was tested using a live/dead staining kit (Live/Dead BacLight™, Invitrogen). They were rinsed again with PBS and incubated for an additional 30 min at 37 °C in a dark box with the staining kit, which included a dye called SYTO^®^ 9, which is used to stain living bacteria and allows for the determination of the number of live bacteria present. The samples were then inspected using a fluorescence microscope, and the images were captured to analyze the coverage of live bacteria on the surface of the samples.

For SEM surface analysis (FEI NOVA NanoSEM 430, FEI Company, Hillsboro, OR, USA), a set of samples with biofilm was separated before staining in the last step. A primary fixative solution and buffer solution were prepared by mixing sucrose, sodium cacodylate, glutaraldehyde, and deionized water. The samples were transferred to a new 24-well plate, and the bacteria were fixed by the primary fixative for 45 min. Subsequently, the samples were cleaned with ethanol and coated with 10 nm gold/platinum before imaging.

## 3. Results and Discussion

**Figure 2:**
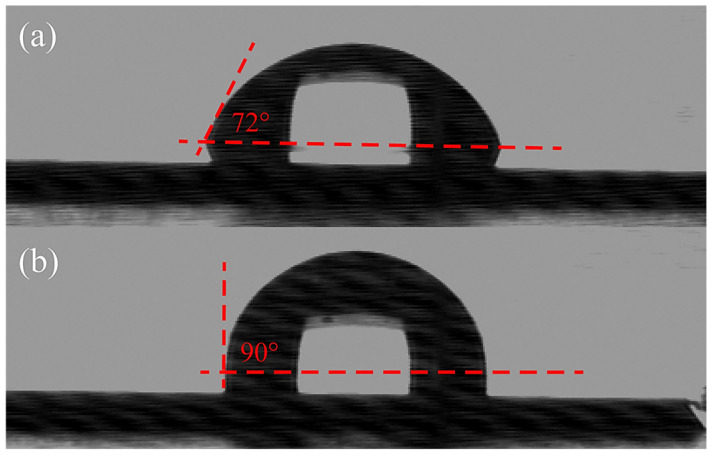
Contact angle images of (**a**) Si and (**b**) quaternized SiCN surface with 5% nitrogen content.

The behavior of a water droplet on the surface of a SiCN substrate before and after the quaternization process is displayed as sessile contact angle images in Figure 2a,b. The decrease in the contact angle on the quaternized surface compared to the untreated surface suggests a successful quaternization process, resulting in an increased hydrophobicity. This enhanced hydrophobicity can be attributed to the presence of extended allyl group chains originating from the surface, resulting from the Menshutkin reaction that converts tertiary nitrogen atoms into quaternary nitrogen atoms. For detailed quantitative information, Table 1 provides the specific average contact angles and standard deviations of Si, SiCN, and QSiCN substrates. Notably, the quaternized SiCN surfaces exhibit contact angles that are 15° to 17° higher than those of the silicon surface, indicating a significant increase in water repellency. Moreover, the differences in contact angles between QSiCN substrates with varying nitrogen contents are minimal, with variances of less than 2°. This suggests that the nitrogen content in SiCN has a negligible influence on the wettability of the QSiCN surfaces.

**Figure 3:**
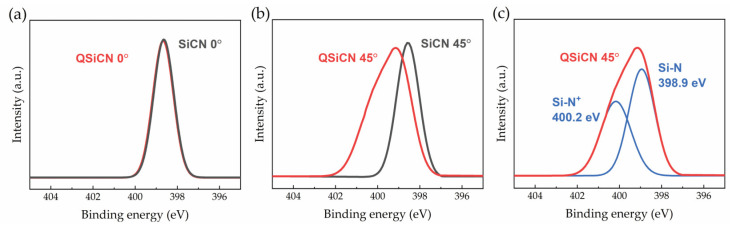
High-resolution XPS spectra of N1 peak analysis of SiCN and QSiCN surfaces with (**a**) 0°- and (**b**) 45°-tilted coating surfaces and (**c**) the peak separation of 45°-tilted QSiCN spectra.

The high-resolution XPS survey scans for the nitrogen 1 s spectra of the SiCN and QSiCN surfaces with a normal angle are shown in Figure 3a. Both SiCN and QSiCN show an identical Si-N main peak at 398.6 eV, indicating no significant differences at a higher X-ray penetration depth. Subsequently, the samples were tilted at 45° with respect to the normal, which reduces the X-ray penetration depth and promotes the signals from the bonding of the surface layer atoms, as shown in Figure 3b. A detailed peak separation for the QSiCN spectra is further shown in Figure 3c. The tilted QSiCN surface spectra reveal a main peak corresponding to Si-N bonds located at 398.9 eV and a satellite peak of the quaternized nitrogen (N+) bound to the silicon at 400.2 eV. This proved the existence of the quaternized nitrogen on the surface of the QSiCN coating and the success of the quaternization process.

**Figure 4:**
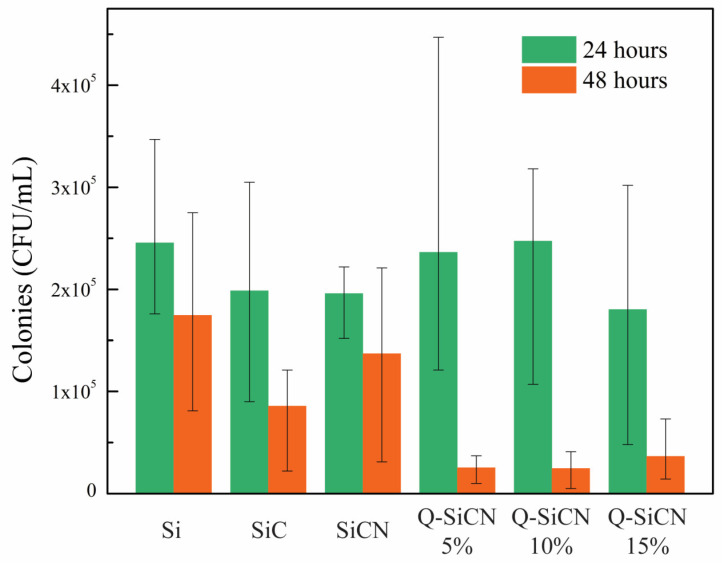
Colony-forming unit (CFU) per mililiter of *S. mutans* culture on Si, SiC, SiCN, and quaternized SiCN substrates with different nitrogen contents after 24 and 48 h of incubation time.

The results of the bacteria culture with *S. mutans,* shown in Figure 4, demonstrate the antibacterial efficacy of the QSiCN surfaces. As a control group, SiC substrates were included, since our previous research established their antibacterial properties. SiCN substrates without quaternization were also included to compare the effects before and after the quaternization process. Three replicates were used for each sample condition, and two sets of incubation times, 24 and 48 h, were employed. To eliminate the possibility of sample contamination by other microorganisms, a blank group with only BHI medium was included as a reference for each condition. During the initial 24 h incubation period, no significant difference in colony count was observed among Si, SiC, SiCN, and any of the QSiCN samples. This phenomenon could be attributed to the resilience of the bacteria, which were freshly cultured 24 h before the experiment and adapted to the new environments. However, after 48 h of incubation, the SiC sample exhibited approximately 50% fewer colonies compared to the Si sample. Notably, all three QSiCN samples with varying nitrogen contents showed significantly fewer colonies, with an over 80% reduction compared to the Si blank control. These findings highlight the remarkable antibacterial effect brought about by the quaternized surface, confirming the antibacterial ability of the SiCN substrates with quaternization. Furthermore, the antibacterial efficiency of the QSiCN samples remains largely unaffected by the three different nitrogen concentrations in the coating, indicating that the nitrogen content has a minimal impact on the bactericidal property of the QSiCN surfaces. This suggests that the quaternization process confers significant antibacterial activity to the SiCN surfaces, regardless of the nitrogen concentration in the coating.

**Figure 5:**
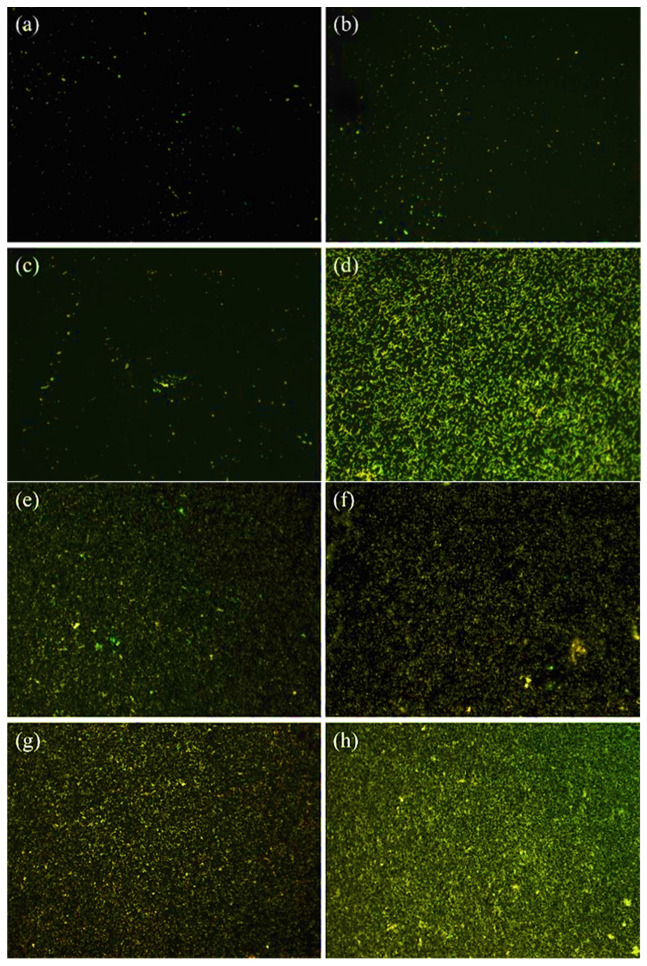
Fluorescence microscopy images of *S. mutans* cultured for three days on quaternized SiCN surface with (**a**) 5%, (**b**) 10% and (**c**) 15% nitrogen content and (**d**) Si as the control group; and seven days culture time on SiCN surface with (**e**) 5%, (**f**) 10% and (**g**) 15% nitrogen content and (**h**) Si as the control group.

The results obtained from the staining assay reveal a noteworthy decrease in the population of viable bacteria on the QSiCN samples as compared to the control group of pure silicon substrates. This is evident from the fluorescence images depicted in Figure 5, where live bacteria are marked in green against a dark background. The coverage of live bacteria on the QSiCN samples is significantly reduced after 3 and 7 days of culture, indicating a bactericidal effect against *S. mutans*. In comparison to the Si sample, the QSiCN samples exhibit visibly less coverage of bacteria on the surface even after 3 days of incubation (Figure 5a). Even after 7 days of incubation, while the bacteria on the Si sample reach near-saturation, the QSiCN samples still demonstrate a distinctly reduced bacterial coverage (Figure 5b). No significant differences were observed with regard to nitrogen concentrations on the QSiCN surfaces.

**Figure 6:**
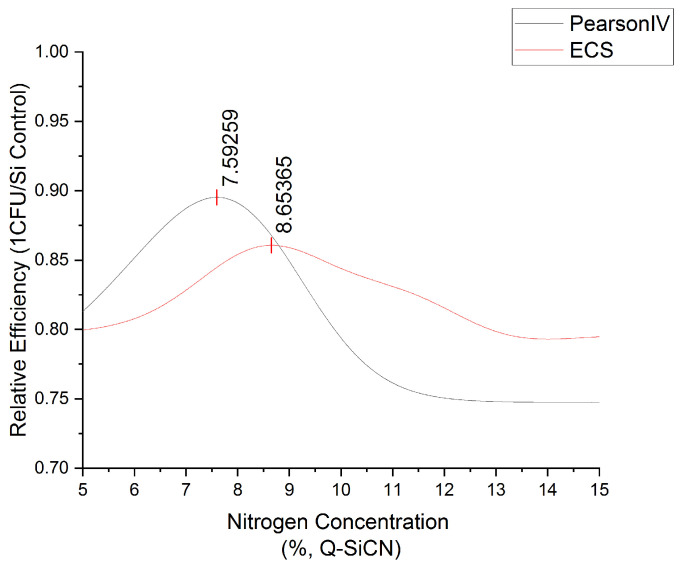
The optimization of nitrogen concentration range in quaternized SiCN using Pearson IV and Edgeworth–Cramer Peak Function.

The Nitrogen% analysis using classical Machine Learning techniques proved unsuccessful due to the extensive variation observed in the dataset, emphasizing the need for a larger number of data points. Nonetheless, a promising breakthrough was achieved by determining the relative efficiency of Q-SiCN in comparison to Si. This was accomplished by employing the formula 1-(CFUexp/CFUcontrol) and integrating it with dose-response equations. Two mathematical equations, namely the Pearson IV and Edgeworth–Cramer Peak Function (ECS), were adapted from chromatography methods and applied to the dataset [46,47]. Although the fit was relatively weak, the results were deemed sufficient for preliminary data-analysis purposes. Refer to the accompanying Figure 6 for visual representation. Based on the available data, the optimal concentration range was estimated to lie between 7.6% and 8.6%. To improve future sample collection, it is recommended that one follow a geometric progression of concentrations, including 1 wt%, 2 wt%, 4 wt%, 8 wt%, and 16 wt%. Further investigations involving a more extensive dataset could facilitate the utilization of machine-learning methods, thereby enhancing the reliability of the findings. This approach holds promise for future research endeavors.

**Figure 7:**
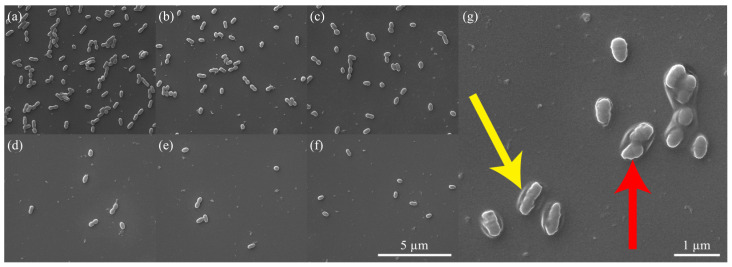
SEM images of the surface of (**a**) Si, (**b**) SiC, (**c**) SiCN, and quaternized SiCN with (**d**) 5%, (**e**) 10% and (**f**) 15% nitrogen content. (**g**) With a larger magnification image of quaternized SiCN with 5% nitrogen content. Note yellow arrow pointing to the ring-shaped pattern and red arrow pointing to irregular outline of bacterial cell.

In order to investigate the underlying factors responsible for the enhanced antibacterial properties of the QSiCN surfaces, SEM images were obtained after fixing the bacterial cells on surfaces that were incubated for 3 days and 7 days under different conditions. Bacterial coverage on non-quaternized surfaces of Si, SiC, and SiCN (Figure 7a–c) demonstrates significantly more bacteria compared to the quaternized surfaces (Figure 7d–f). Upon applying a higher magnification, an intriguing observation was made in Figure 7g, which depicts the 5% nitrogen-containing QSiCN surface. The SEM image revealed a ring-shaped pattern in the surrounding area, along with an irregular outline of the bacterial cell, indicating ruptures in the bacterial membrane. This suggests that the bacterial cells on the quaternized SiCN surface underwent membrane damage, leading to cell termination on the surface of the substrate [15,30,48]. This finding provides valuable insight into the potential mechanism underlying the enhanced antibacterial effect of the QSiCN surfaces. 

## 4. Conclusions

The results of this study demonstrate the successful quaternization of SiCN surfaces, resulting in an increased hydrophobicity and significant antibacterial properties against *S. mutans*. The contact angle measurements revealed that the QSiCN surfaces exhibited higher contact angles compared to untreated Si surfaces, indicating an increased water repellency. The XPS analysis indicated successfully quaternized nitrogen atoms on the SiCN surface. The antibacterial efficacy of the QSiCN surfaces was confirmed through bacterial culture experiments, demonstrating significant reductions in colony counts compared to control groups. The staining assay further revealed a reduced bacterial coverage on the QSiCN surfaces after 3 and 7 days of culture, indicating a bactericidal effect. SEM images provided insights into the potential mechanism underlying the enhanced antibacterial effect, suggesting membrane damage of the bacterial cells on the QSiCN surfaces. Overall, these findings highlight the potential of QSiCN surfaces for applications in antibacterial coatings, with further investigations warranted to fully elucidate the underlying mechanism.

## Figures and Tables

**Table 1:** Sessile contact angle of Si, SiCN and quaternized SiCN substrate with various nitrogen contents.

Sample	Contact Angle (°)
Si	72 ± 2
SiCN with 5% nitrogen content	75 ± 1
Quaternized SiCN with 5% nitrogen content	90 ± 1
Quaternized SiCN with 10% nitrogen content	88 ± 2
Quaternized SiCN with 15% nitrogen content	87 ± 1

## Data Availability

Data available upon request.
